# BioMOBS: A multi-omics visual analytics workflow for biomolecular insight generation

**DOI:** 10.1371/journal.pone.0295361

**Published:** 2023-12-14

**Authors:** Dries Heylen, Jannes Peeters, Jan Aerts, Gökhan Ertaylan, Jef Hooyberghs

**Affiliations:** 1 Theory Lab, Data Science Institute (DSI), Hasselt University, Diepenbeek, Belgium; 2 Flemish Institute for Technological Research (VITO), Mol, Belgium; 3 Data Science Institute (DSI), Hasselt University, Diepenbeek, Belgium; 4 Visual Data Analysis Lab, Department of Biostystems KU Leuven, Leuven, Belgium; Boyce Thompson Institute, UNITED STATES

## Abstract

One of the challenges in multi-omics data analysis for precision medicine is the efficient exploration of undiscovered molecular interactions in disease processes. We present BioMOBS, a workflow consisting of two data visualization tools integrated with an open-source molecular information database to perform clinically relevant analyses (https://github.com/driesheylen123/BioMOBS). We performed exploratory pathway analysis with BioMOBS and demonstrate its ability to generate relevant molecular hypotheses, by reproducing recent findings in type 2 diabetes UK biobank data. The central visualisation tool, where data-driven and literature-based findings can be integrated, is available within the github link as well. BioMOBS is a workflow that leverages information from multiple data-driven interactive analyses and visually integrates it with established pathway knowledge. The demonstrated use cases place trust in the usage of BioMOBS as a procedure to offer clinically relevant insights in disease pathway analyses on various types of omics data.

## Introduction

In precision medicine, the rapidly expanding amount of multi-omics data requires concerted efforts on data integration. Currently, biological networks are widely applied to represent multi-omics data [1]. Still, it remains a challenge to generate a fine-grained focus within a holistic network view of (disease-related) molecular biology [2, 3]. We present a workflow that meets this challenge by allowing us to highlight interesting subsets from a plethora of data-derived interactions while integrating the biological predefined knowledge about these subsets. This enables comprehensive exploratory analysis that can provide an alternative view on predefined disease pathways, exposing more detailed intra- and inter-omics interactions.

**Fig 1:**
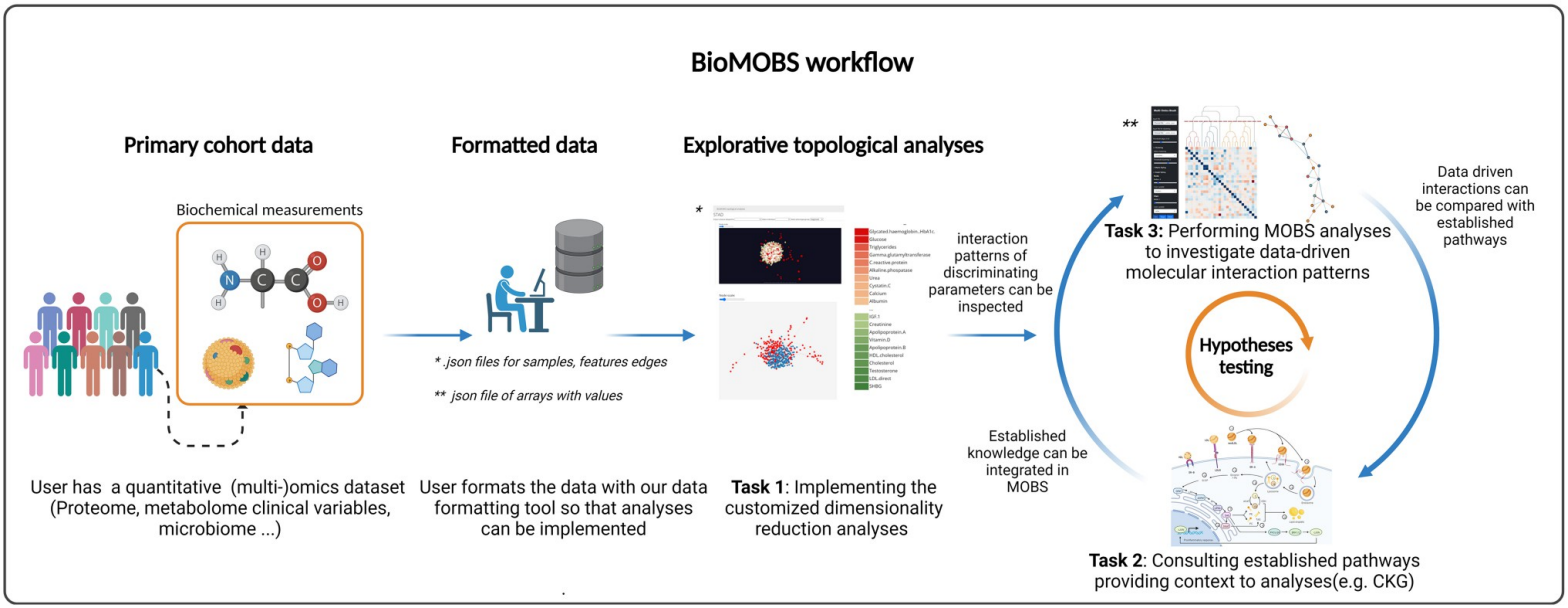
BioMOBS workflow overview. For a pathology of interest, task 1 demarcates the primary scope of interest. A data driven topological image is obtained in task 1. Whereas established biomolecular pathway knowledge is obtained in task 2. Task 3 (MOBS) integrates the data driven and literature based information, allowing clinically relevant analyses. Users can implement multiple types of omics data simultaneously in one data load. http://creativecommons.org/licenses/by/4.0/ [5].

The proposed workflow (Fig 1) consists of three tasks. A data driven topological image is obtained in task 1, whereas established bio-molecular pathway knowledge is obtained in task 2. A multi-omics brush for subgraph visualization (MOBS) is the final cornerstone. MOBS was designed as an in-house visualization tool and a preliminary version was presented as ongoing work to the data visualization community earlier [4].

The three tasks are elaborated and as a proof of principle BioMOBS’ workflow is applied on a (type 2 diabetes) subset of UK biobank data.

## Results

**Fig 2:**
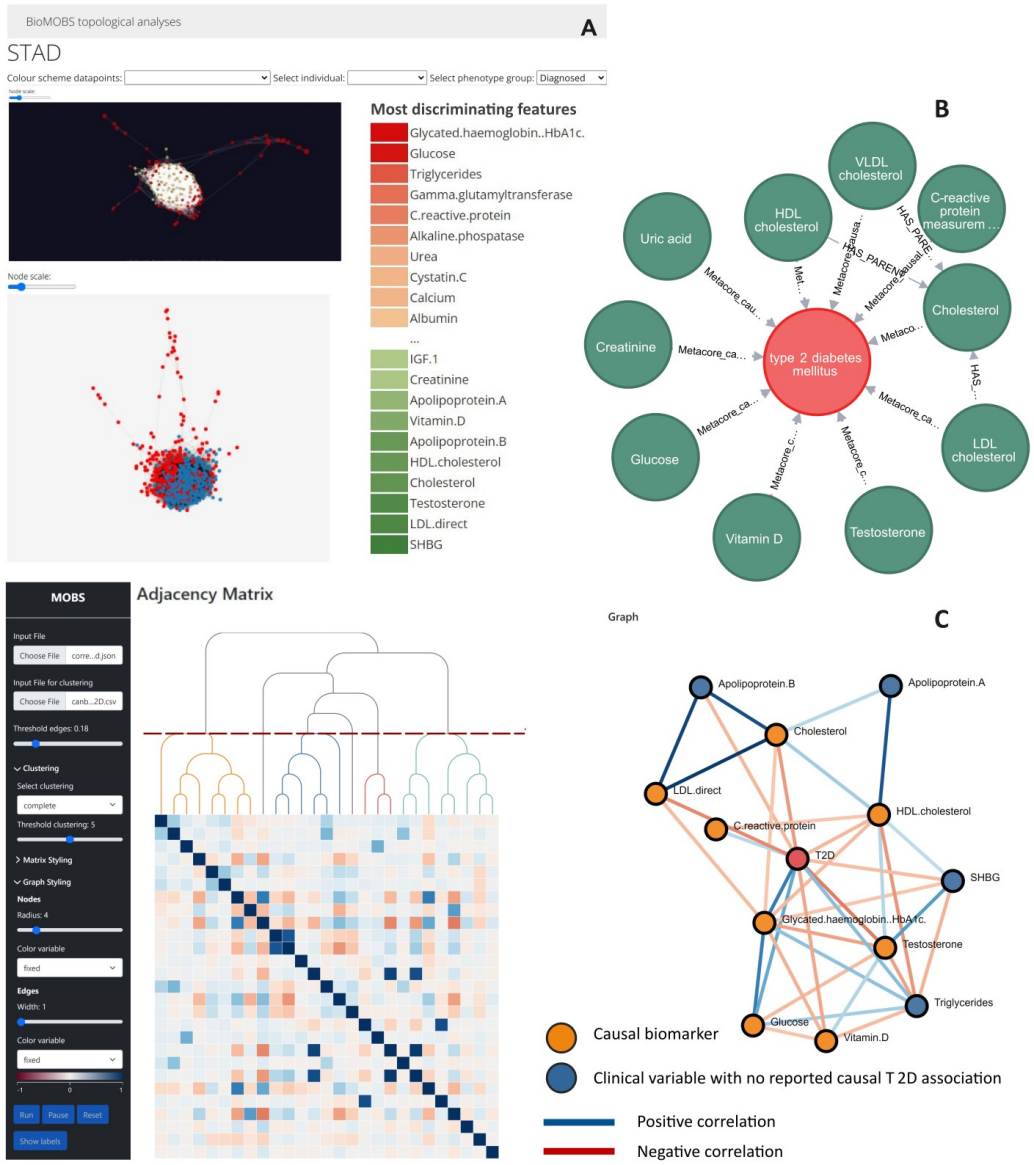
Use case application of BioMOBS workflow for type 2 diabetes mellitus (type 2 diabetes). All shown analyses are performed on UK Biobank data containing 28 clinical biochemistry measurements of 402 individuals half of whom have a type 2 diabetes diagnosis. (N = 402, 201 type 2 diabetes patients, 201 healthy patients). Healthy patients have no self-reported illnesses and all 28 biochemistry measurements are within normal population range intervals. A) Customized topological dimensionality reduction analyses with individuals as nodes. Nodes of the selected type 2 diabetes diagnosed individuals are red in the node-link visual (2D and 3D view). In the feature loading bars green vs. red bars indicate high vs. low abundance of the parameter in the selected group (diagnosed individuals) compared to the non-selected group. Here HDL cholesterol is for example low in the diagnosed group compared to the undiagnosed, healthy group. B) Explanatory CKG network that shows the previously reported causal knowledge with regard to type 2 diabetes for all 28 investigated bio-molecules. C) MOBS on a correlation adjacency matrix where each cell represents a weighted correlation between the biomolecular parameters. Clustering is performed here on a canberra distance measure. Edge threshold set on 0.28. Nodes are coloured based on information from Fig 2B.

The first task of this workflow checks, for a given set of parameters, if and how the molecular profile of diagnosed individuals is different from that of healthy individuals. Fig 2A shows a Simplified Topological Abstraction of Data (STAD) analysis. STAD generates an abstract representation of the analyzed samples (both healthy individuals & type 2 diabetes individuals) by creating a graph where each node represents a sample and links are drawn between samples that are similar [6]. The visualisation has an interactive drop down menu to select a category of samples or a single sample (selected samples are indicated in red (Fig 2A)). The feature values of the selected samples are then compared with the rest of the population on a per feature basis with the following formula:

Formula 1 [7]:

$$\begin{array}{c} \delta =\frac{\text{median}\;\text{selected}\;\text{samples}\;\text{—}\;\text{median}\;\text{non-selected}\;\text{samples}}{\text{Interquartile}\;\text{range}\;\text{(all}\;\text{samples)}} \end{array}$$

Where a high absolute value for “delta” indicates that the median value of the corresponding feature is highly different in the selected group of individuals compared to the unselected individuals. This results in the feature loading bars on the right side where the 20 most discriminating features are displayed (Fig 2A). The distinct type 2 diabetes patient data cloud differs from healthy individuals by means of e.g. higher Hba1c, glucose and triglyceride values (red) and e.g. lower HDL values (green) (Fig 2A). This exploratory first task gives a broad data derived indication of the parameters affected by the disease. The second task focuses on integrating established biomolecular pathway knowledge to complement (or substantiate) the information retrieved from task one. For the application use case a molecular reference map (Fig 2B), relevant for the UK Biobank set of clinical parameters, shows which of the ‘inspected’ biochemical parameters are reported in literature to be causally associated with type 2 diabetes. The Clinical knowledge graph (CKG) environment was in this application complemented with information derived from Metacore [8]. CKG is an open-source platform for the mining and integration of knowledge from various biomedical data sources [5]. One of CKG’s advantages is that users can easily modify interactions and add sources to a local version of the platform in a graph database environment. This advantage allows the integration of alternative (licensed) databases, such as e.g., Reactome, Metacore, KEGG, or String/Stitch pathways, on a study/disease-specific basis to obtain a complete reference pathway map [9–12]. Thus, in essence, task two of our workflow provides a scientifically validated ‘state of the art network’ for a pathology of interest. Fig 2B displays this with clinical biochemistry parameters for the type 2 diabetes use case.

In the workflow’s final task, the various functionalities of MOBS can be utilized for pathway exploration. Taking into account the potential features of interest that are substantiated in the first two tasks, Fig 2C demonstrates an analysis example for this dataset. All analysis settings can be manually adjusted within MOBS when the data is loaded. The order of the adjacency correlation matrix from the molecular parameters of our type 2 diabetes dataset was rearranged based on hierarchical clustering. The threshold clustering level was set to five, indicated by the red dotted line, to determine the colouring of different dendrogram sub-trees. The type 2 diabetes diagnoses node was included here as a molecular feature and thus treated numerically. No sub-graph was brushed yet in this example, since a node-link edge threshold setting of 0.18 resulted in a network containing many of the type 2 diabetes features of interest identified in task 1 & 2 (see S1 Fig for brush example). The nodes are coloured based on the metadata provided in task 2. The edge colour is an edge weight that corresponds with the colour of the cells in the matrix. As indicated, most biochemical features in the resulting node-link diagram (Fig 2C) are established causal biomarkers for type 2 diabetes. As type 2 diabetes is a well-described disease on a clinical biochemistry level, the identification of established causal biomarkers validates BioMOBS as a workflow to correctly identify essential features in a disease pathway. The same benchmarking was obtained for hypertension (S1 Fig). However, from this type 2 diabetes data perspective, features such as triglycerides and sex hormone-binding globulin (SHBG) are placed on the same level as those causal biomarkers (Fig 2C). This output can be considered as a hypothesis generated by the workflow. The node-link diagram in Fig 2C tells us that these features are the only parameters correlating that strongly with the disease, that are not previously reported as a causal type 2 diabetes biomarker. This finding does not necessarily imply causality. Instead, at this stage, it should be seen as a potential insight generated by the workflow, rather than an absolute proof. For validation purposes it would therefore be of interest to verify and potentially reassess the role of those features in type 2 diabetes established pathways (available at this point in a CKG graph database environment after task 2). This process of extensive biological verification is needed to explore pathway specific biomolecular mechanisms that support the findings of the workflow. However, that exercises would take us out of the scope of this methodological report.

**Fig 3:**
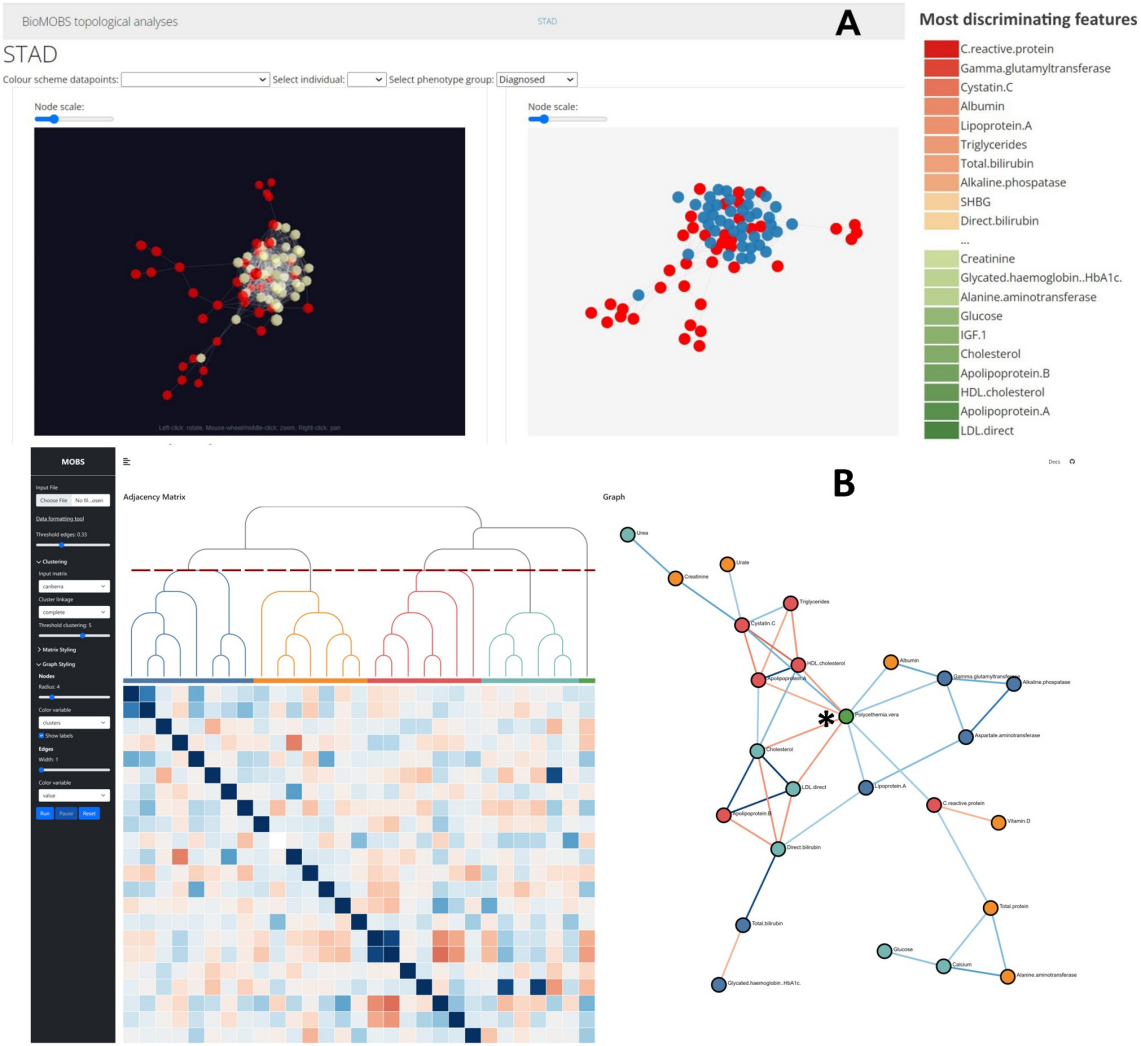
Use case application of BioMOBS workflow for polycythemia vera (pol.vera). All analyses are performed on UK Biobank data containing 28 clinical biochemistry measurements of 80 individuals of which half have a pol.vera diagnosis. (N = 80, 40 pol.vera patients, 40 healthy patients). Healthy patients have no self-reported illnesses and all 28 biochemistry measurements are within normal population range intervals. A) Customized topological dimensionality reduction analyses where nodes of the pol.vera diagnosed individual are red in node-link visual; green vs red bars = high vs low abundance of parameters in the selected group compared to the non-selected group. B) MOBS on a correlation adjacency matrix where each cell represents a weighted correlation between the parameters. Clustering performed is based on a Canberra distance measure. Edge threshold set on 0.32. Nodes coloured based on the dendrogram colours. There are no causal clinical associations known for pol. vera (disease node indicated with an asterisk).

The technical versatility of MOBS as a visualisation tool is larger than apparent from the type 2 diabetes example alone. A detailed user manual is available in S4 Fig for users to understand all functionalities. We will highlight some specific (interactive) capabilities in more detail as they provide valuable analysis options. In Fig 2C interactions between features were correlations, however, the tool accepts any interaction data such as topological interaction patterns (S2 Fig), molecular interactions, or binary interactions (specified as JSON formatted graph objects). This enables users to analyze their datasets based on the various types of data-interactions they contain. An important interactive feature of the tool revolves around brushing (i.e. using the mouse cursor, clicking and dragging across the adjacency matrix), to extract sub-parts of the graph for on demand exploration (S1 Fig). Tooltips are available in the node-link diagram displaying information on all variables when hovering over the diagram. The show labels check box in the sidebar enables users to get all labels of the nodes so that the entire network is immediately annotated. Node color can be customized based on metadata to integrate pre-established knowledge (Fig 2C). Alternatively, the node color can also be customized based on the dendrogram tree so that it matches with their corresponding sub-tree colour on the matrix view (Fig 3). If users want to explore one specific sub-tree in more detail, clicking the colored bars underneath the dendrogram (Fig 3) automatically renders that sub-tree as a node-link diagram. This way these colored bars enable efficient exploration of parameters of interest without the need for users to manually brush the exact region of interest on the adjacency matrix.

## Discussion

With BioMOBS, we made an effort to close the gap between the visualization community and precision medicine. Although similar designs using the principle of brushing on the adjacency matrix have been introduced before [13–15], BioMOBS is unique since it is specifically customized for the exploration of multi-omics data (S3 Fig) in biomolecular disease pathways and integrated in a disease contextualization workflow. The combination of interactive visualisation options in MOBS can be seen as a collection of the most pertinent elements of the current state of the art in network visualisation, tailored into a systems biology analysis tool. The strength of BioMOBS is that users can interactively boil the data down to the most remarkable patterns of interest while maintaining the context of how these specific patterns relate to the rest of the dataset, as well as to the established pathway knowledge. An additional benefit is the fact that all interactions are visible on the adjacency matrix which allows the user to zoom in on any region of interest, providing both overview and detail on demand. Despite these advantages, analyses might still benefit from some user guidelines. Therefore we suggest to start the initial scope of analyses within MOBS with a focus on interactions related to the features demarcated in task 1 and task 2 (Fig 2A and 2B). That way, users can assess to what extent a disease, and its biomarkers, interact with either known or unknown biochemical features. This strategy is also opted for in the type 2 diabetes application.

The presented analyses for type 2 diabetes with clinical biochemistry data can analogously be performed with any (mix of) alternative omics data. Dependent on the composition of the features and the disease of interest, slightly different MOBS visualization approaches might be opted for (Fig 3 & S2 Fig). In line with that, an important remark is that the clinical context of type 2 diabetes is already well-described. The results here show that BioMOBS positively identifies several biomarkers of type 2 diabetes. Remarkably, LDL values show to be negatively associated with type 2 diabetes (Fig 2A & 2C) even though high LDL is historically broadly known to cause bad metabolic health [16]. This supports more recently published findings about type 2 diabetes research that reports low LDL cholesterol with increased type 2 diabetes risk [17, 18]. Applying this workflow to omics types that are not yet established in clinical practice, or on diseases that are to a more limited extent described, will allow users to explore a wide range of undescribed hypotheses. Fig 3 is an example of such. There are no established clinical biomarkers for polycythemia vera, yet both Fig 3A as Fig 3B provide data derived insights that can be further investigated for biological verification in disease pathways.

The task 3 MOBS toolset provides a clear visual overview of up to 500 molecular parameters, but above that, the computational load slows down the visualisation. Therefore algorithms like mapper [19], which allow the extraction of global features from a dataset with higher dimensions, will be considered in future usage. Another remark concerns the brush methodology that is designed to only select contiguous cells. To deal with that, different distance measures and cluster algorithms are available in order to generate various types of clustering that reorder the matrix. Additionally the edge threshold option, allows the user to focus on interactions annotated with a specific weight. That way subclusters of strong interactions can for example be obtained regardless of their contiguously in the matrix (Fig 2C). In the future, alternative analyses strategies can be applied in the form of matrix reordering algorithms [20, 21].

## Conclusion

BioMOBS is a workflow that leverages information from multiple data-driven interactive analyses and visually integrates it with established pathway knowledge. The demonstrated use cases place trust in BioMOBS’ usage as tool to perform clinically relevant pathway analyses.

## Materials and methods

**Fig 4:**
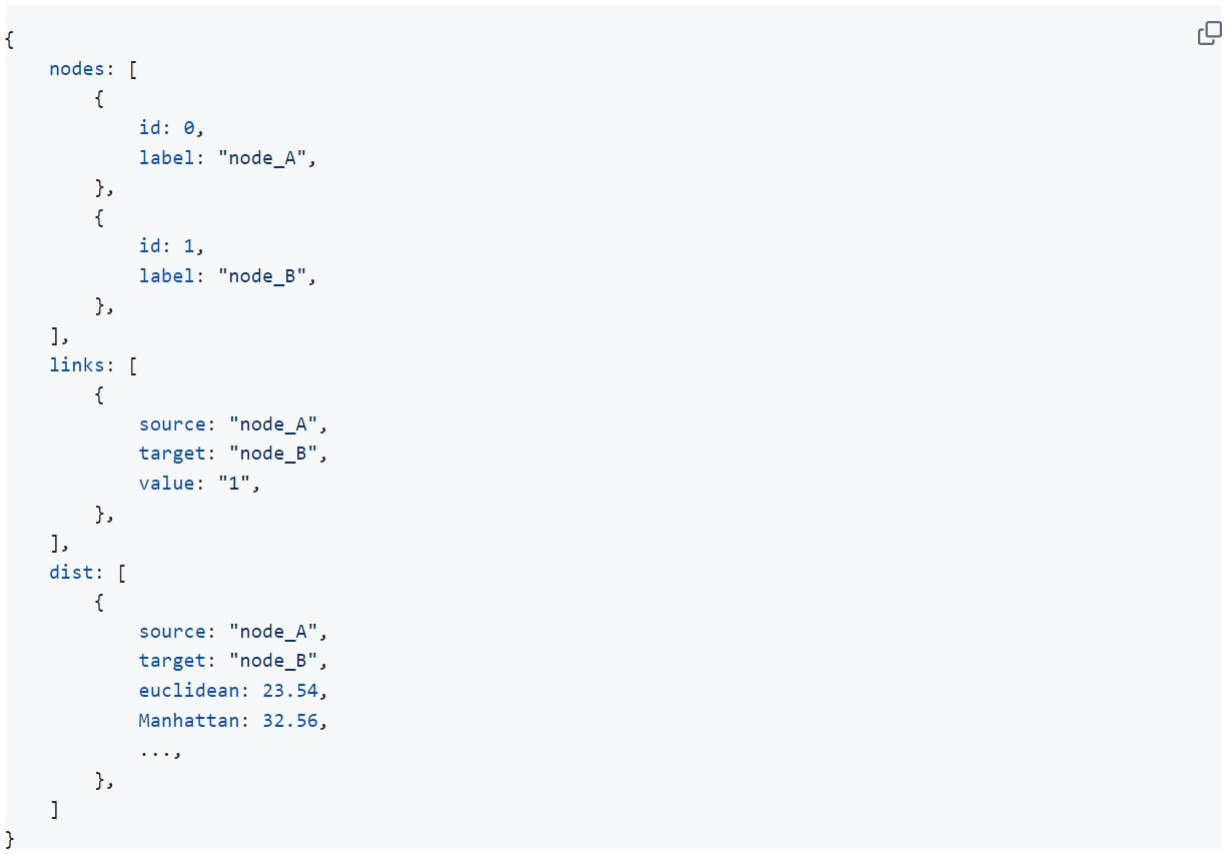
Data requirements. The input data for Task 3 needs to be of ‘.json’ format and should contain at least a ‘nodes’ and ‘links’ array, potentially added on with a ‘dist’ array to perform the clustering functions. This complete format is automatically generated when using our data formatting tool. Users can always include other distance metrics when preferred.

The topological visualization source code is publicly available at (https://github.com/driesheylen123/BioMOBS). This design reads data from an ArangoDB back end and renders a Svelte & D3 visualization. MOBS is a web-application also developed in Svelte (https://svelte.dev/), using the D3.js library [22], and can either run locally after installation or can be used from the online hosted version on vercel (https://mobs.vercel.app//). The source code is publicly available (https://github.com/driesheylen123/Multi_omics_exploration. Detailed instructions on how to use and install the tool are provided in the README.md file on Github and at the “Docs” button in the webtool. Formatted example (Fig 4) data and a data formatting tool (https://biomobs.shinyapps.io/shiny/) to demonstrate and facilitate the functionality of the tool are also available. The selected dataset(s) contains the clinical biochemistry parameters that UK Biobank measures. Samples were included based on reported disease cases in the male population of the UKB cohort. Besides the samples of diagnosed people, also healthy individuals where included, i.e. if they had no reported diagnoses and all their biochemistry measurements lay within the normal population reference interval (PRI) [23]. The data for which the demonstrated findings are obtained is available upon request for researchers via the access management system of UKB. Analogue dummy data sets are available on our github directories. Established pathway knowledge used in task 2 is gathered from CKG and the Metacore database. CKG is an open source platform that integrates and mines knowledge from multiple biomedical databases [5]. Metacore is an extensive manually created database where biological effects and interactions that are currently substantiated by biomedical research, are accumulated [8].

For the topological analyses visualization (task 1), three basic.json files (describing samples, features and edges) should be provided to the source code. Test data is available in the GitHub repository.

For MOBS (task 3), one input file should be uploaded, its format is described in Fig 4. Clicking on the “Docs” button in the upper right corner of the web-tool, renders test data. This test data is also available in the github repository. Metadata variables, such as node annotations, or edge weights indicating confidence measures for specific interaction can be easily added in the original input file to the users’ liking (for description see Docs button).

In order to facilitate these data requirements, a data formatting tool was designed (https://biomobs.shinyapps.io/shiny/). Out of a standard matrix with features as columns and samples as rows this formatting tool generates the required data formats for the different visualisations. This allows users to not only load the test data to have a first impression of the workflow but also format their own data in a fast way with very limited effort. For MOBS analyses (Task 3) for example, the formatting tool provides users with the ‘input file’, which is a json file containing the nodes and their quantified reciprocal interactions, as well as a distance matrix in which the distance between the nodes of the visual is computed. An example matrix as described above can be downloaded within the formatting tool where it can serve as input data for the different conversions. Users immediately see a preview of the appropriate data format (Fig 4) in the formatting tool to get a grasp of how the workflow reads your data.

## Supporting information

S1 Fig Use case application of BioMOBS workflow for hypertension. All shown analyses are performed on UK Biobank data containing 28 clinical biochemistry measurements. of 400 individuals from which half of them have a hypertension diagnosis. (N = 400, 200 hypertension patients, 200 healthy patients). Healthy patients have no self-reported illnesses and all 28 biochemistry measurements are within normal population range intervals. A) customized topological dimensionality reduction analyses where nodes of the healthy diagnosed individual are red in node-link visuals (2D and 3D); green vs red bars = high vs low abundance of parameters in the selected group compared to the non-selected group. c) MOBS on a correlation adjacency matrix where each cell represents a weighted correlation between the parameters. Clustering performed using the Canberra distance measure. Edge threshold set on 0.31. Nodes coloured based on information from 2B. The numbers 0 and 1 behind the node labels indicate to which part of the dendrogram the nodes belong (blue or yellow).
(TIF)

S2 Fig As indicated MOBS can handle any type of ‘json’ formatted graph object. To illustrate this here MOBS is loaded with topological edges as interactions. Links are drawn by STAD, only between samples that are similar. When a link is indeed present it was annotated with the original distance value between the two nodes that connect through that link/edge.
(TIF)

S3 Fig An example of a multi-omics correlation analyses on an in-house test dataset. Node colours indicate different omics types. Any combination of quantitative data types can be concatenated and imported into MOBS.
(TIF)

S4 Fig Detailed instruction manual for the different visualization features available in MOBS https://mobs.vercel.app/. (TIF)
