# Supplementary figures and images for: BioMOBS: A multi-omics visual analytics workflow for biomolecular insight generation
Source: PLoS One. 2023 Dec 14;18(12):e0295361. doi: 10.1371/journal.pone.0295361 (PMC10721075; doi:10.1371/journal.pone.0295361)

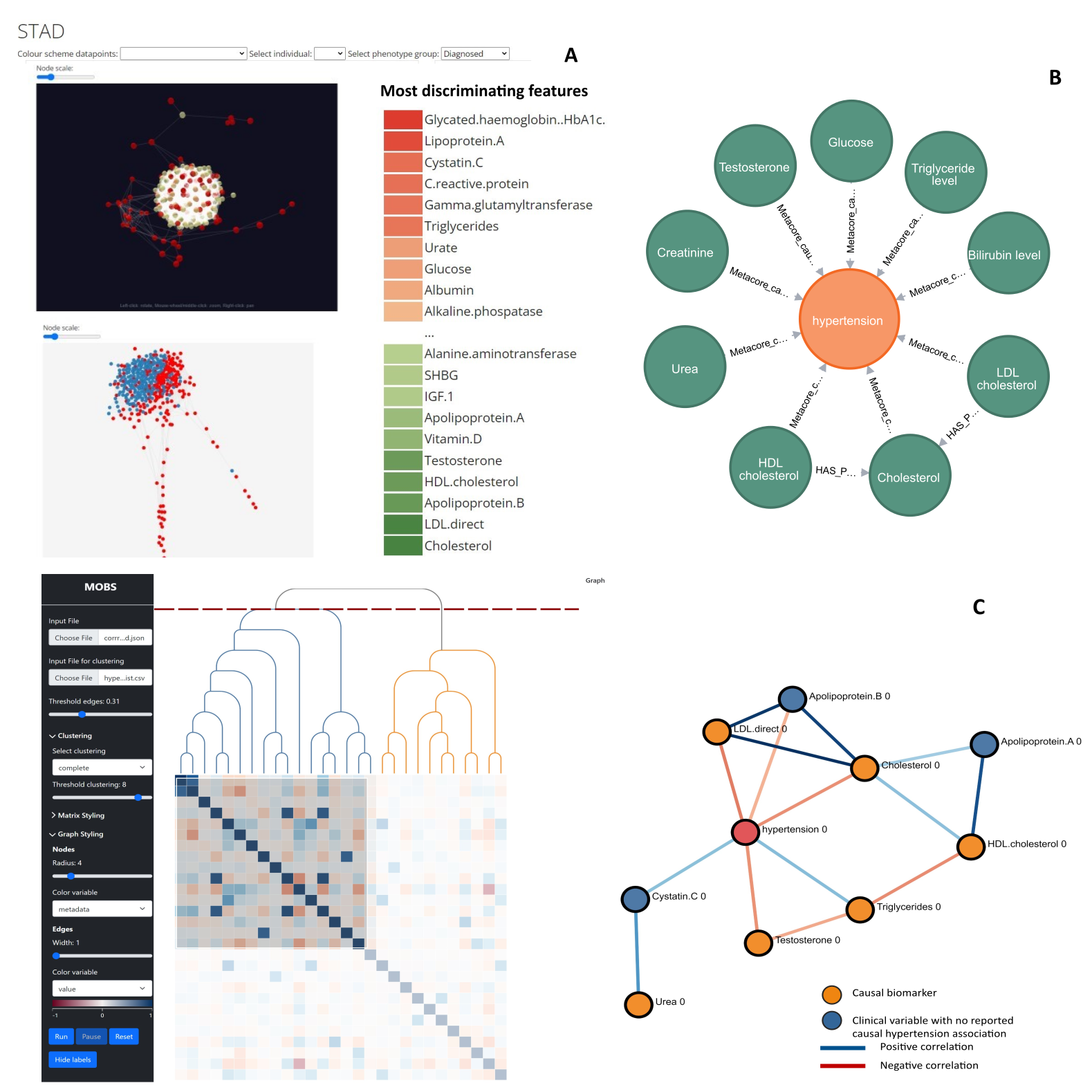

Supplement: S1 Fig — All shown analyses are performed on UK Biobank data containing 28 clinical biochemistry measurements. of 400 individuals from which half of them have a hypertension diagnosis. (N = 400, 200 hypertension patients, 200 healthy patients). Healthy patients have no self-reported illnesses and all 28 biochemistry measurements are within normal population range intervals. A) customized topological dimensionality reduction analyses where nodes of the healthy diagnosed individual are red in node-link visuals (2D and 3D); green vs red bars = high vs low abundance of parameters in the selected group compared to the non-selected group. c) MOBS on a correlation adjacency matrix where each cell represents a weighted correlation between the parameters. Clustering performed using the Canberra distance measure. Edge threshold set on 0.31. Nodes coloured based on information from 2B. The numbers 0 and 1 behind the node labels indicate to which part of the dendrogram the nodes belong (blue or yellow). (TIF) [file pone.0295361.s001.tif]

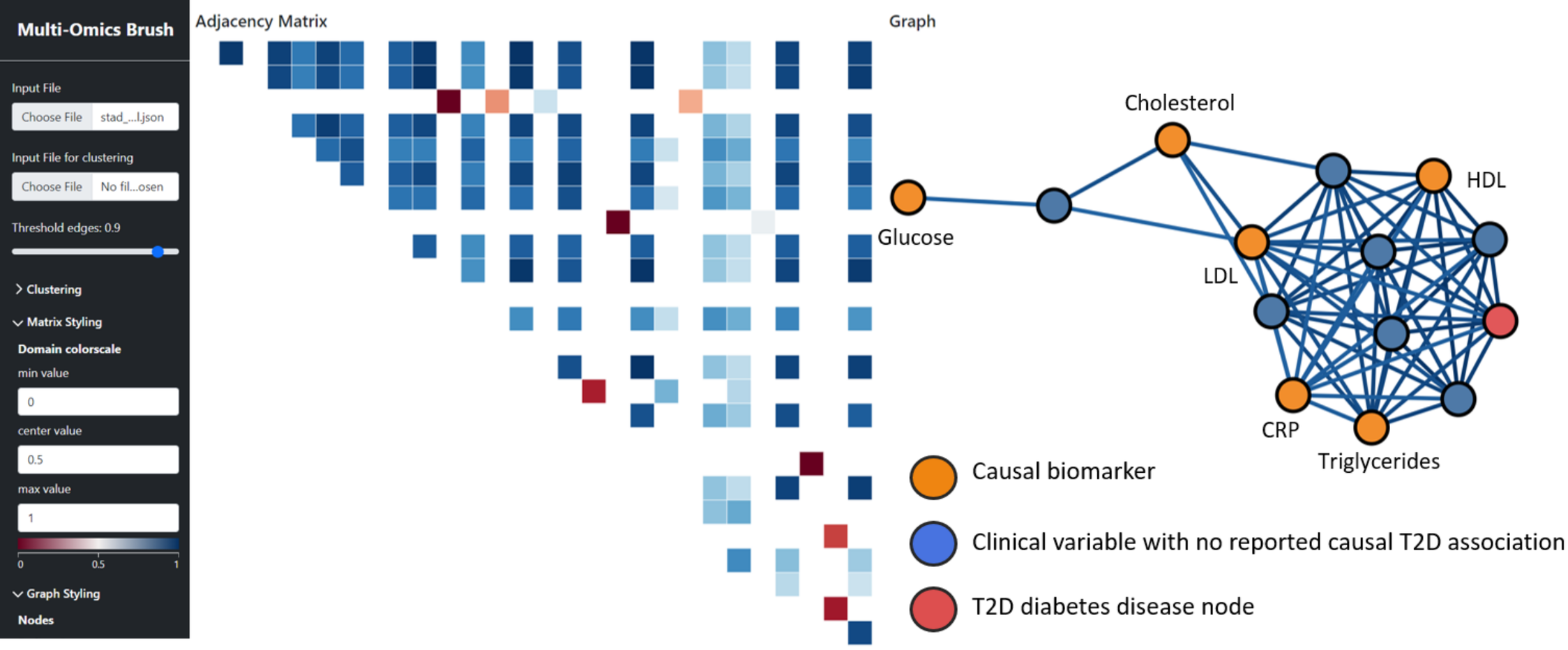

Supplement: S2 Fig — To illustrate this here MOBS is loaded with topological edges as interactions. Links are drawn by STAD, only between samples that are similar. When a link is indeed present it was annotated with the original distance value between the two nodes that connect through that link/edge. (TIF) [file pone.0295361.s002.tif]

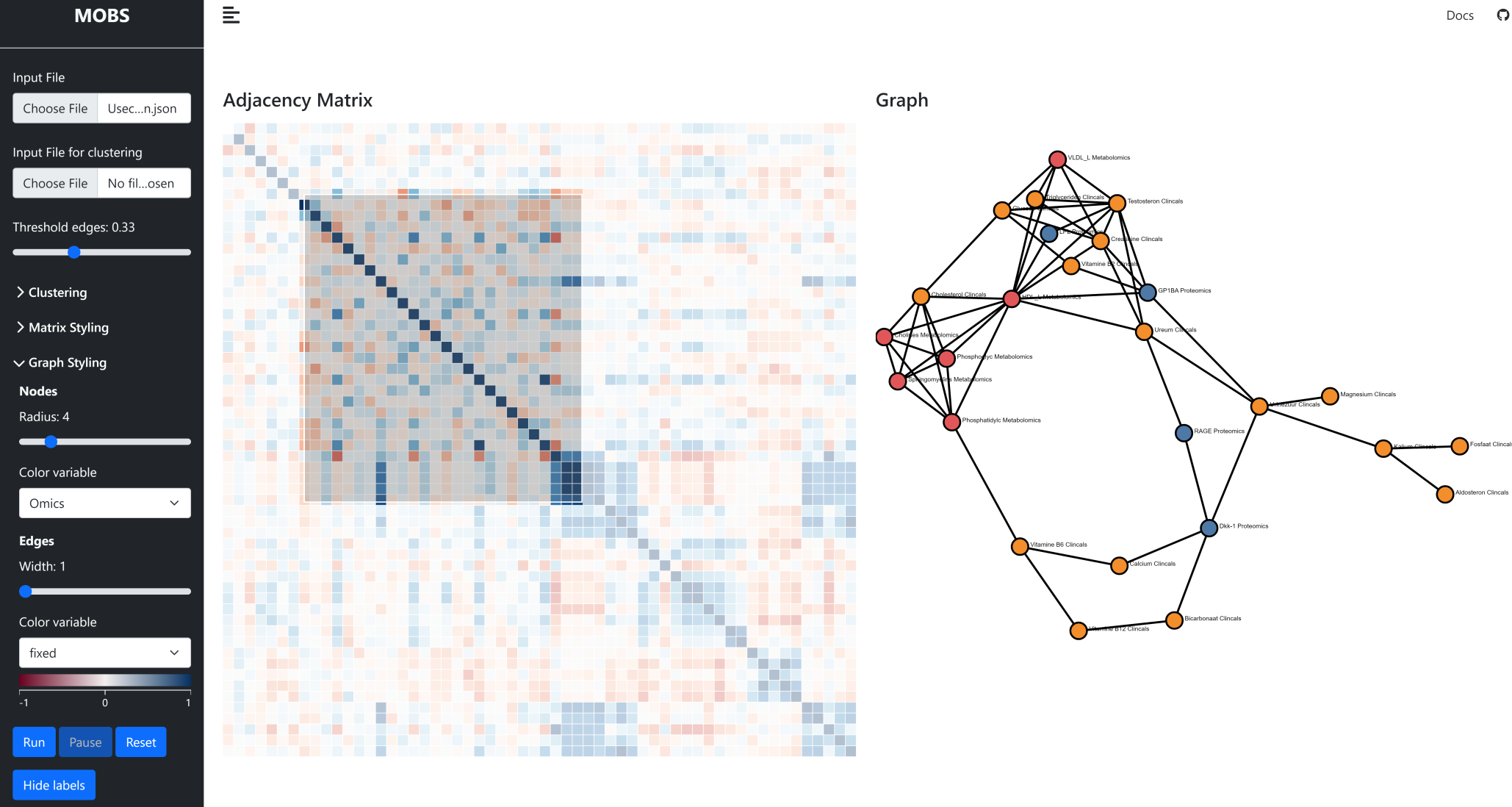

Supplement: S3 Fig — Node colours indicate different omics types. Any combination of quantitative data types can be concatenated and imported into MOBS. (TIF) [file pone.0295361.s003.tif]

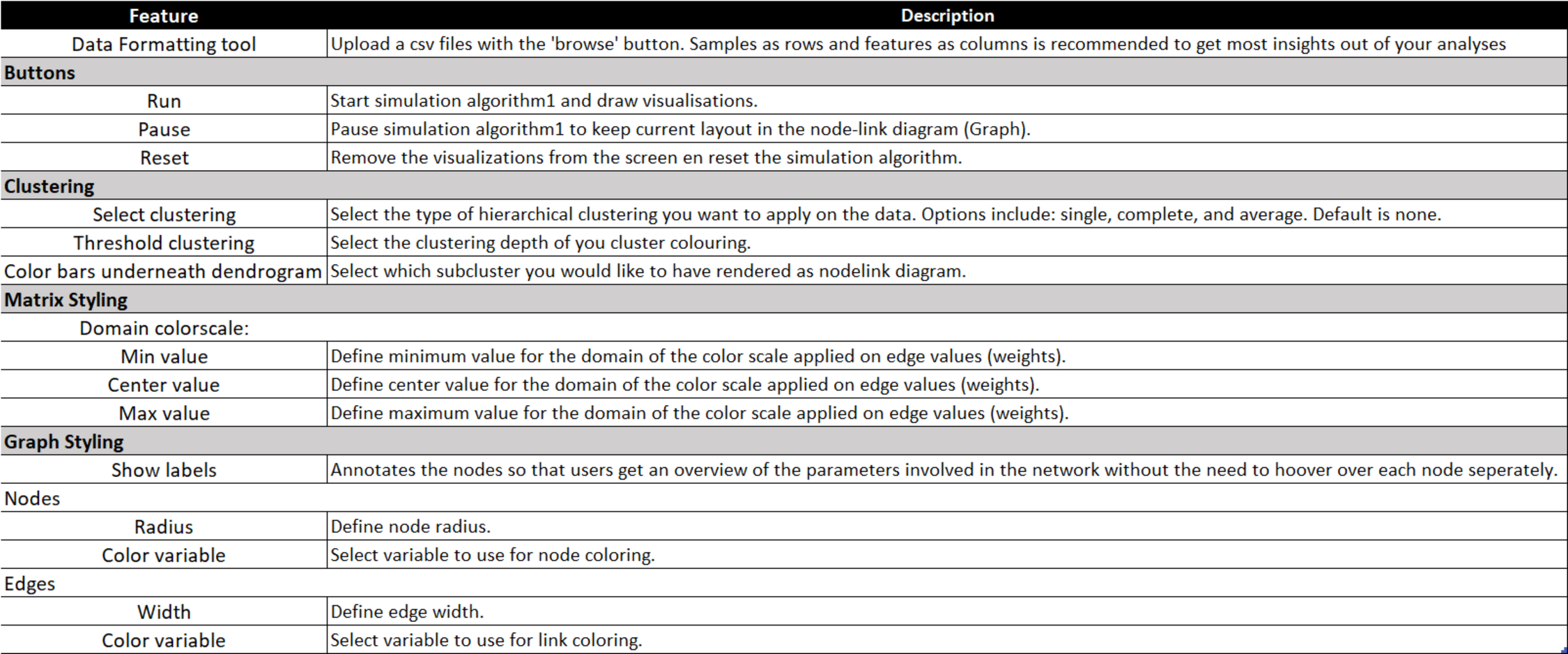

Supplement: S4 Fig — (TIF) [file pone.0295361.s004.tif]
